# A Voxel-Wise Meta-Analysis on the Cerebellum in Essential Tremor

**DOI:** 10.3390/medicina57030264

**Published:** 2021-03-14

**Authors:** Ioannis Mavroudis, Foivos Petrides, Eleni Karantali, Symela Chatzikonstantinou, Jack McKenna, Alin Ciobica, Alin-Constantin Iordache, Romeo Dobrin, Constantin Trus, Dimitrios Kazis

**Affiliations:** 1 Laboratory of Neuropathology, Electron Microscopy First Department of Neurology, Aristotle University, 54124 Thessaloniki, Greece; i.mavroudis@nhs.net (I.M.); f_petridis83@yahoo.gr (F.P.); 2 Leeds Teaching Hospitals, Leeds LS97TF, UK; jackmckenna@doctors.org.uk; 3 Institute for Research of Alzheimer’s Disease, Other Neurodegenerative Diseases and Normal Aging, Heraklion Langada, 54123 Thessaloniki, Greece; 4 Third Department of Neurology, Aristotle University of Thessaloniki, 54124 Thessaloniki, Greece; lena.kar@outlook.com (E.K.); melina.chatzik@gmail.com (S.C.); dimitrios.kazis@gmail.com (D.K.); 5 Department of Biology, Faculty of Biology, Alexandru Ioan Cuza University, B dul Carol I, No 11, 700506 Iasi, Romania; alin.ciobica@uaic.ro; 6 Center of Biomedical Research, Romanian Academy, B dul Carol I, No 8, 700506 Iasi, Romania; 7 Faculty of Medicine, “Grigore T. Popa”, University of Medicine and Pharmacy, Strada Universitatii 16, 700115 Iasi, Romania; romeodobrin2002@gmail.com; 8 Department of Morphological and Functional Sciences, Faculty of Medicine, Dunarea de Jos University, 800008 Galati, Romania

**Keywords:** essential tremor, voxel-based, meta-analysis, cerebellum

## Abstract

*Background and Objectives*: Essential tremor is a chronic progressive neurological condition. The clinical presentation of essential tremor is heterogeneous and includes involuntary tremor on hands or arms and progressively on head, jaw, and voice. More extensive and complex symptoms may also be noticed in several patients. Many studies have been carried out to identify biomarkers to help the diagnosis, however, all the efforts have not shown any substantial results yet. *Materials and Methods:* Here, we aimed to perform a voxel-based meta-analysis using a dedicated cerebellar mask to clarify whether the results from the previous studies are robust and have any clinical significance. We included studies with a total of 377 essential tremor patients and 338 healthy control individuals. *Results:* A significant regional decrease in the volume of the gray matter was detected in the right cerebellar hemispheric lobule IV/V, and in the cerebellar vermic lobule IV/V. *Conclusions:* This is the first study focused on the cerebellum and using a specific cerebellar mask, which increases the sensitivity. It showed regional statistically significant changes that could not be seen in the whole-brain analysis.

## 1. Introduction

Essential tremor is a neurological disorder with heterogeneous clinical presentation. Patients with essential tremor exhibit involuntary tremor on hands or arms. Some patients may also have tremor of the head, the jaw, and voice [1,2,3,4]. A number of patients may develop complex symptomatology [5,6,7,8,9,10]. According to the revised diagnostic criteria, the diagnosis of essential tremor demands isolated action tremor on both upper limbs with a minimum duration of three years, with or without tremor in the head, voice, and lower limbs [5]. Although additional neurological findings—such as dystonia, ataxia, and/or parkinsonism—might be absent, mild memory impairment and impaired tandem gait can be present, and these patients are diagnosed with essential tremor plus syndrome [5].

The neuropathological and neuroimaging features of essential tremor have been studied in multiple studies; however, the pathophysiological mechanism is not yet clearly understood. The cerebellum is one of the brain areas that has been investigated in essential tremor. A dysfunction of the corticothalamo-olivo-cerebellar pathways, a dysfunction of the GABAergic network, and cerebellar degeneration are widely accepted as some of the most prevailing theories [6].

Despite the efforts to identify essential tremor (ET) biomarkers, the results as yet are limited; the development of neuroimaging techniques, however, and, specifically, voxel-based morphometry (VBM), has shown promising, although heterogeneous findings. Several studies found a gray matter decrease in ET [7,8,9,10,11,12,13], while other studies reported no significant differences [14,15,16,17,18,19,20,21], and further studies have shown gray matter increase [22,23,24].

Two recent meta-analyses showed alterations in the gray matter in different brain areas in ET; however, the results lack reliability because of the significant heterogeneity and publication bias [25,26]. Here, we conducted a voxel-wise meta-analysis on the cerebellar alterations in essential tremor. We used the effect size-based signed different mapping (ES-SDM), which is a quantitative voxel-based meta-analytic tool for neuroimaging that has been used extensively in a number of neurodegenerative and neuropsychiatric conditions [27,28,29,30]. We also used a cerebellar brain mask (SUIT) to identify changes in the region of interest (cerebellum) because this method is more sensitive than whole-brain analysis [31].

## 2. Methods

### 2.1. Data Sources, Study Inclusion, and Data Extraction

The present voxel-wise meta-analysis was carried out following Preferred Reporting Items for Systematic Reviews and Meta-Analyses (PRISMA) guidelines. We searched the online database of PubMed with the keywords (“essential tremor” OR “ET”) and (“Voxel-based” OR “morphometry” OR “VBM”). The BrainMap database was also searched for VBM data on ET. All the included studies compared the gray matter changes between ET patients and healthy individuals and reported the results in Talairach of Montreal Neurological Institute space. Exclusion criteria were no control group and no detailed coordinates for the contrasts.

For each study, 2 independent researchers extracted the number of participants, as well as additional demographics including the age, duration of disease, additional symptoms where available, and the peak coordinates.

For the voxel-wise meta-analysis, we used the ES-SDM software (Version 6.11). The ES-SDM software, also known as Seed-based d Mapping, is a statistical software for meta-analyses on the differences in structural or functional differences of the brain and can be applied to studies with Functional magnetic resonance imaging (fMRI), Voxel-based morphometry (VBM), Diffusion Tensor Imaging (DTI) or Positron emission tomography (PET). The SDM software recreated an effect-size map of the regional grey matter volume differences using a meta-analytical random-effects model where each study’s weight was calculated by the sample size, intra-study variability, and between-study heterogeneity. We set the full width at half-maximum at 20 mm, and the statistical threshold as *p* < 0.005. These values have been shown by previous studies to have a great control for false positives, as well as for the optimization of the balance between sensitivity and specificity [32]. We also did Jackknife sensitivity analyses to assess the reliability of the results by repeating the analysis and omitting one study each time to check the results’ reliability. We further used a random effects model with Q statistics to reveal significant unexplained between-study variability within our results [29,32].

Finally, we performed meta-regression analyses to examine effects of the age, the illness duration and severity and the presence or not of head tremor, on the results of the meta-analysis [33].

### 2.2. Assessment of Risk of Bias

For the evaluation of risk of bias, we used the Cochrane Risk of Bias tool, which requires the studies to be rated as low risk, unknown risk, and high risk on the following: random sequence generation, allocation concealment, blinding of participants and personnel, blinding of the outcome, incomplete outcome data, selective reporting, and other sources of bias. The Cochrane Risk of Bias tool for the visualization of the results uses a traffic light system. High risk is represented as red with the minus sign, unclear risk as yellow with the minus sign, and low risk as green with the positive sign.

We visually inspected the funnel plots and carried out Egger’s tests to investigate possible publication bias.

## 3. Results

**Figure 1:**
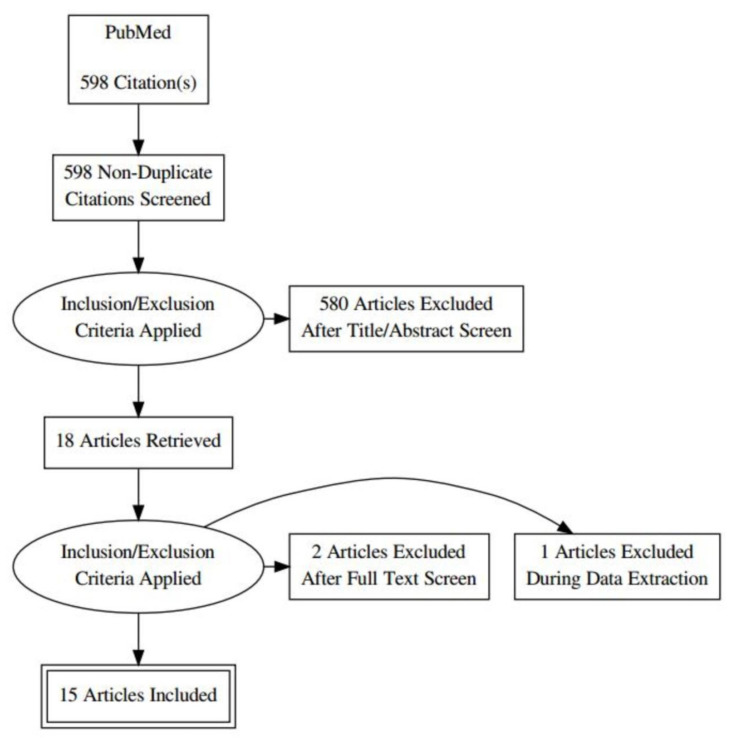
Preferred Reporting Items for Systematic Reviews and Meta-Analyses (PRISMA) flow diagram showing the flow of information through the different phases of the selection process, mapping out the number of records found, included, and excluded.

We initially found 598 studies in the online databases. After excluding duplicates, reviews, and meta-analyses, only 16 studies met the inclusion criteria. In one of them, the coordinates were not available, and therefore 15 studies were finally included in the present meta-analysis, with a total of 377 ET patients and 338 NC (Figure 1). The mean age for ET patients was 55.6 years and the mean disease duration was 20 years. Two studies (14, 21) were performed with a 1.5 Tesla MRI scanner, and the rest of them with a 3.0 Tesla MRI machine. Fourteen studies reported their results in MNI coordinates and one in Talairach coordinates.

### 3.1. Summary of the Included Studies

Buijink et al. [18], in their study, investigated the volumetric changes of the cerebellum in three different sub-studies. The first one included 36 familial, propranolol-sensitive essential tremor patients and 30 healthy controls, the second included 9 sporadic essential tremor patients against 9 healthy controls, and the third included 45 essential tremor patients against 8 familial cortical myoclonic tremor patients and 39 controls. They reported no significant changes in the cerebellar volume on essential tremor patients compared to controls [18]. Cao et al. [24] compared the gray matter changes in 17 patients with essential tremor under the age of 60 years, with 17 age- and gender-matched healthy controls. They reported a significant bilateral increase of the cerebellum in patients with essential tremor, and no significant correlation between these changes and the age, gender, tremor duration, family history, tremor severity, and mini-mental state examination. Cameron et al. [8], in their study on 47 essential tremor patients and 36 controls, described no difference in the cerebellum between the groups of the study, while Daniels et al. [14] also failed to demonstrate considerable changes in the cerebellar volume between 27 patients with essential tremor and 27 controls. Klein et al. [15] conducted a voxel-wise analysis and found white matter changes in essential tremor patients, but they reported no significant change in the volume of the cerebellum. Fang et al. [16], using resting-state functional MRI and voxel-based morphometry, found differences in anterior and posterior bilateral cerebellar lobes, but no difference in the volume of the cerebellum. Nicoletti et al. [17], using VBM and functional MRI on 32 patients and 12 healthy controls, reported functional but not volumetric changes in the cerebellum. Archer et al. [20] investigated the functional and volumetric changes in 19 patients with essential tremor and 18 control subjects, and they did not find significant changes on the volume of the cerebellum in the ET group. Benito-Leon et al. [7] recruited 19 essential tremor patients and 20 age-matched controls for their study on the structural changes of the brain in essential tremor, and they found significant gray matter changes on both cerebellar hemispheres and other brain areas. Bagepally et al. [11] investigated the grey matter in the cerebrum and the cerebellum in 20 essential tremor patients and 17 matched control subjects, reporting scattered areas of cerebral and cerebellar atrophy in ET patients compared to healthy individuals. On another study, Cerasa et al. [12] reported a mild atrophy of the anterior cerebellar cortex in 14 patients with essential tremor compared to 23 controls matched for age and gender, while Bhalsing et al. [13], using 25 essential tremor patients and 25 matched controls, also reported gray matter volume loss in the cerebellum on both anterior and posterior lobes on the patient group. Quatrrone et al. [21] investigated the presence of gray matter abnormalities in patients with essential tremor. They recruited 50 patients with familial essential tremor and 32 healthy controls. They reported substantial atrophy of the vermis in patients with essential tremor involving the head compared to controls. Gallea et al. [22] similarly reported loss of the volume of the gray matter in the cerebellar cortex, and more specifically in the lobule VIII in 19 individuals diagnosed with essential tremor in comparison to 19 controls, whereas Lin et al. [23] also reported certain volumetric changes in the cerebellum of 10 patients with essential tremor, in comparison to 12 age-matched controls.

### 3.2. Quality of the Selected Studies

**Figure 2:**
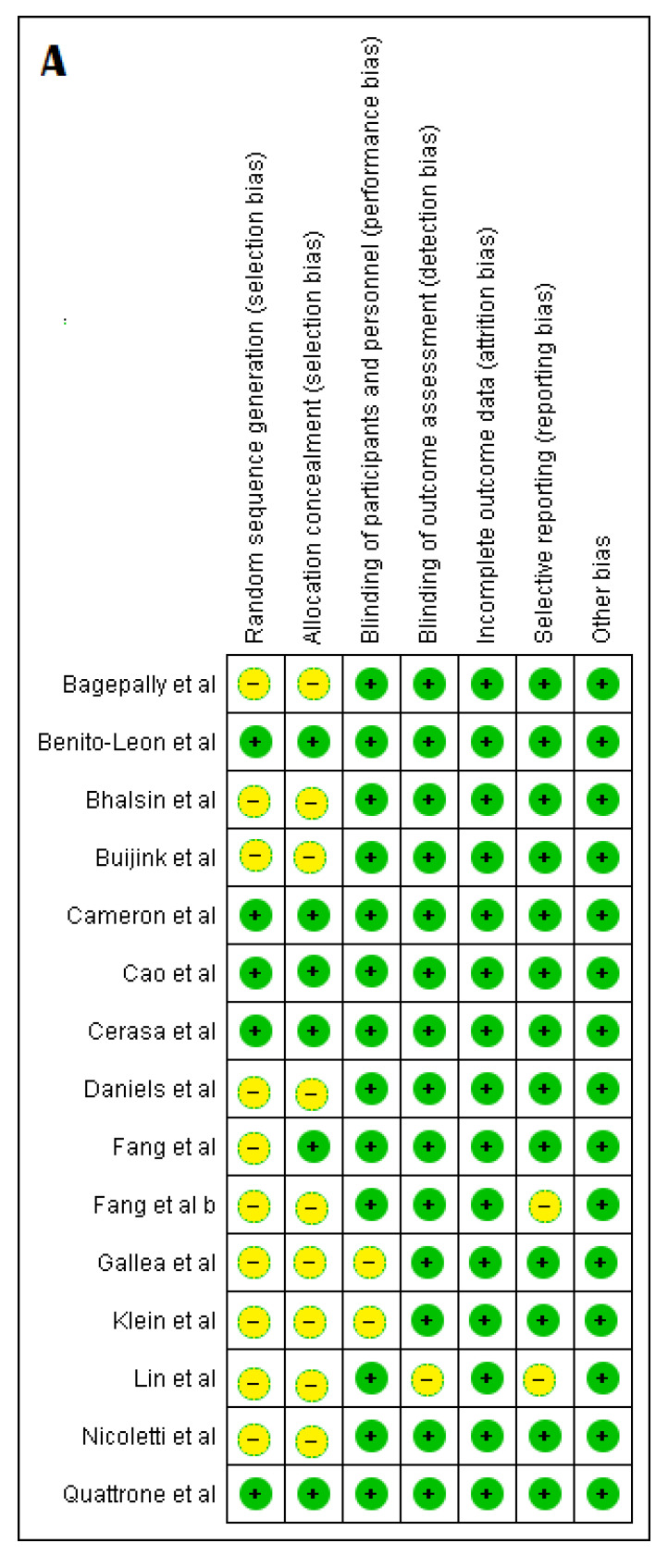

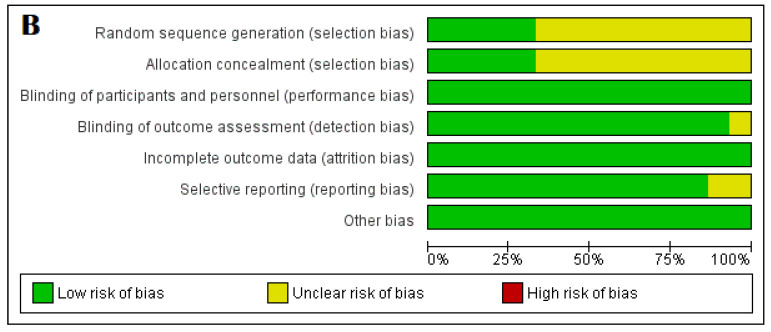
(**A**) Risk of Bias assessment; (**B**) Risk of Bias summary.

Evaluation of the included studies with the Cochrane Risk of Bias Tools showed that most of the studies were rated as having an unclear risk in the domains of random sequence generation and allocation concealment, which could be indicative of a bias in the selection process (Figure 2A,B). No other significant biases were noticed on the evaluation of the studies.

### 3.3. Regional Differences in Gray Matter Volume

**Figure 3:**
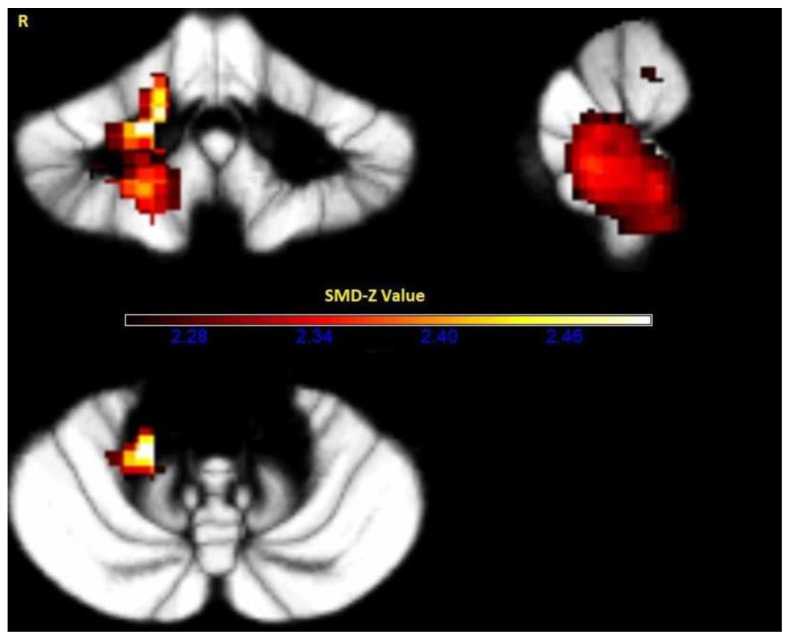
Gray matter decreased in the cerebellar vermis and the right cerebellar hemisphere of the essential tremor patients compared to normal controls. SDM = Seed-based d Mapping.

A substantial decrease in the gray matter was detected in the right cerebellar hemispheric lobule IV/V, SMD-Z = −2.590, *p* < 0.0001, number of voxels = 18414, and in the cerebellar vermic lobule IV/V, SMD-Z = −1.301, *p* = 0.0008, number of voxels = 45 (Figure 3).

### 3.4. Heterogeneity and Risk of Bias

**Figure 4:**
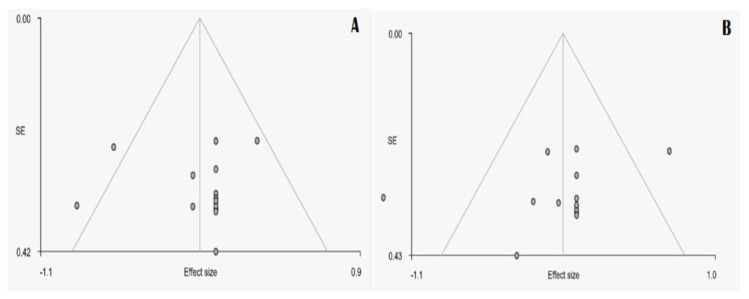
Funnel plots of the statistically significant results of the meta-analysis for the cerebellar vermis (**A**) and the cerebellar hemispheres (**B**). The horizontal axis represents effect sizes; the vertical axis represents the standard errors.

No significant asymmetry was found on funnel plots, and furthermore Egger’s test was not significant for both areas that showed significant gray matter changes between groups. Heterogeneity in published studies was significant (*Q* = 31.992, *p* = 0.00004) (Figure 4A,B).

### 3.5. Jackknife Analysis

Jackknife analysis confirmed the reliability of the findings, especially the gray matter decrease in the cerebellar hemispheres, which was replicable in 15 out of 15 studies. The changes in the vermic lobule IV/V were replicable in 12 out of 15 studies.

### 3.6. Meta-Regression

Meta-regression analysis revealed a positive relationship between disease duration and the left cerebellar hemispheric lobule VI (MNI: −28, −62, −24; z = −2.28; *p* = 0.00002, number of voxels: 518), age and the right cerebellar hemispheric lobule VI (MNI: 14, −50, −1; z = −2.72; *p* < 0.0001; number of voxels = 776), and severity of tremor and an increase in the gray matter at the left cerebellar hemispheric lobule IV/V (MNI: −24, −36, −2; z = 1.02; *p* < 0.00001; number of voxels = 1005).

## 4. Discussion

The current study is not the first Voxel-wise meta-analysis in ET. Still, it is the first focused on the cerebellum using a specific cerebellar mask, which increases the sensitivity of caching regional changes that could not be seen in the whole-brain analysis.

Essential tremor has been previously related to inferior olivary nucleus; however, accumulating evidence shows that it is mainly connected to the cerebellum and the locus coeruleus [34,35,36,37]. The pathophysiology is not known as of yet; however, neuropathological and neuroimaging studies have demonstrated interesting, although heterogeneous findings. Amongst the most critical findings are specific morphological changes of the Purkinje cells of the cerebellar cortex [34], axonal alterations to the olivocerebellar climbing fibers, changes on glutamate transporters, and Lewy bodies in the locus coeruleus [38].

Numerous neuroimaging studies have also underlined cerebellar contribution in essential tremor pathophysiology. Shin et al. reported a non-statistically significant loss of the volume of the cerebellum and a significant reduction in the cerebellar vermis volume in 39 ET patients compared to normal controls [39]. An additional VBM study on 50 ET patients showed noticeable atrophy of the vermis of the cerebellum in patients with tremor on both arms and head in comparison to controls. In contrast, patients with tremor on the arms only exhibited a non-statistically important loss of the volume of the vermis [21]. Further studies have shown regional atrophy of the cerebellar cortex, but with no overall cortical atrophy [14], and widespread cerebellar changes, except without atrophy in specific areas of the cerebellum [7,23]. A recent VBM study on 18 ET patients and 20 healthy controls matched for age and gender by Cao et al. revealed a number of differences in the gray matter and an expansion of the volume of the grey matter bilaterally in the cerebellum. They suggested that these regional gray matter changes could represent functional or compensatory changes [24].

Han et al., in a voxel-wise meta-analysis based on 10 studies with 241 ET patients and 213 healthy individuals, reported a loss of the gray matter volume in different cerebral areas but not a significant difference in the cerebellum [25]. In comparison, Luo et al., in another voxel-wise meta-analysis with 16 studies on 387 ET patients and 355 controls, reported no significant regional gray matter changes [26].

In the present study, using a dedicated cerebellar mask, we found a significant gray matter volume reduction on the right cerebellar hemispheric lobule IV/V and the cerebellar vermic lobule IV/V, and these findings seemed to be reliable and were replicable in Jackknife analysis, with low risk of publication bias and high heterogeneity. It is important to emphasize that the findings of the present study were in the right cerebellar hemisphere and a part of the vermis. This lateralization is difficult to explain, yet it could be related to the divergence of features of patient samples. Previous studies have linked the asymmetry in the pathological changes in essential tremor with a possible asymmetry in the symptomatology [40]. Indeed, Louis et al. (2014) investigated the pathological changes of the cerebellum in 28 ET patients and found a strong correlation between lateralization of the tremor and neuropathological findings in the cerebellum [40]. The findings of the present study are in accordance with these of neuropathological studies on the cerebellum [40].

The heterogeneity found in the present and other neuroimaging studies on essential tremor has been attributed and may reflect variations in sample sizes, demographics, and clinical features of ET patients.

We also found a positive relationship between the patients’ age and a decrease in the gray matter of the right hemispheric lobule VI and disease duration and the right hemispheric lobule VI. Interestingly we found a relationship between tremor severity and an increase in the gray matter of the left hemispheric lobule IV/V.

The gray matter decrease that we found here is unknown in terms of whether it is causatively related to the symptomatology and thus could be the aftermath of long dysfunction of these areas. At the same time, the increase in the left hemispheric lobule IV/V and tremor severity has been suggested to be a compensatory change.

### Limitations

The studies included in the present meta-analysis were conducted before the establishment of the new classification of essential tremor. They did not divide the patients into essential tremor and essential tremor plus groups, and patients with different types of tremor were not categorized into different types.

Unfortunately, the high heterogeneity makes voxel-based morphology a less reliable method for the discrimination between ET patients and controls.

Further studies that will take into account the lateralization of tremor would be useful in order to investigate if the volumetric changes noticed in the cerebellum that are related to the clinical presentation. Last but not least, additional studies that will consider the new classification of tremor and will apply the new diagnostic criteria for essential tremor and categorize patients on the basis of both clinical phenotypes and the etiology needed in order to clarify neuroimaging changes in essential tremor.

## 5. Conclusions

The present study showed that the cerebellum undergoes certain volumetric changes in essential tremor patients in comparison to normal controls; however, the high heterogeneity makes the result less reliable. Additional studies will apply the revised diagnostic criteria for essential tremor and will consider the 2016 classification needed to clarify the cerebellar changes in essential tremor.

## Data Availability

All the current data is available on request from the authors.
